# Laparoscopic Subtotal Splenectomy: A Feasible Option in the Treatment of Splenic Metastasis

**DOI:** 10.1245/s10434-024-16549-2

**Published:** 2024-11-22

**Authors:** Cristina Izquierdo, Alberto García-Picazo, Juan Pablo Rodríguez, Anna Navarro, Greta Donisi, Eduardo Luque, Benedetto Ielpo, Fernando Burdío, Patricia Sánchez-Velázquez

**Affiliations:** 1 Division of Hepato-Biliary and Pancreatic Surgery, Department of Surgery, University Hospital del Mar-IMIM (Hospital del Mar Medical Research Institute), Barcelona, Spain; 2 Universitat Pompeu-Fabra, Barcelona, Spain

## Abstract

**Introduction:**

Laparoscopic subtotal splenectomy (LSS) is a procedure that helps avoid the consequences of asplenia. Given the spleen’s importance and functionality, there may be specific indications and patient conditions in which partial splenectomy is beneficial. This case report aims to clarify the indications for LSS and outline the surgical technique.

**Methods:**

This multimedia article provides a comprehensive overview of the surgical procedure, emphasizing key steps to avoid postoperative bleeding and complications.

**Results:**

The patient was hospitalized for 3 days without any complications observed. Postoperative hemoglobin levels were 11.2 g/dL, with no signs of anemia. The patient was discharged with a follow-up visit after 2 weeks, showing no evidence of postoperative complications. The anatomopathological study revealed a nodular area with extensive tumoral necrosis.

**Discussion:**

LSS is a safe surgical option that might help to mitigate the consequences of total splenectomy. Innovative technology in hemostasis, such as COOLINGBIS^©^ (a monopolar electrosurgical electrode designed for hemostatic sealing and coagulation) can help manage this risk. Identifying the vascular pedicle via computed tomography scan is crucial to prevent unexpected bleeding.

**Conclusions:**

In selected cases, LSS is a feasible and safe procedure when performed with appropriate laparoscopic equipment and by experienced surgeons.

**Supplementary Information:**

The online version contains supplementary material available at 10.1245/s10434-024-16549-2.

Total splenectomy has become the preferred method for treating various splenic diseases; however, this surgery is known to be associated with complications arising from infectious or embolic processes, such as subphrenic abscess formation, portal thrombosis, pulmonary hypertension, thrombocytosis, thromboembolism, and post-splenic sepsis. Infectious complications are the most common and usually begin postoperatively, with an incidence of up to 40%,^1,2^ and the risk persists throughout life, as severe post-splenectomy sepsis syndrome may occur in 5–7% of cases.^3^ Surgical removal of the spleen results in impaired clearance of both intracellular and extracellular antigens from the bloodstream, reduced macrophage function, and altered immunoglobulin (Ig) M production. This leads to a diminished specific response to polysaccharide antigens, resulting in both specific and non-specific immune deficiencies, and a heightened susceptibility to severe bacteremia caused by encapsulated agents.^4^

Although performed infrequently, partial splenectomy might help to avoid the previously mentioned consequences of asplenia. Given the spleen’s crucial role and functionality, it has been identified as beneficial for certain indications and patient conditions, such as cystic/hydatid lesions, benign tumors, hematological diseases, partial splenic rupture, and even malignant lesions.^3,5–8^ This approach has been suggested to help preserve some spleen function while reducing postoperative hospital stay and morbidity,^9^ as described in the systematic review published by Liu et al.^5^ The aim of this multimedia article was to provide a comprehensive overview of the laparoscopic subtotal splenectomy (LSS) surgical technique, emphasizing key steps to avoid postoperative bleeding and complications.

## Case Report

We present a case of a 68-year-old man with no known drug allergies, a former smoker, and a former drinker. His most relevant medical history includes non-ischemic dilated cardiomyopathy with severe ventricular dysfunction and moderate mitral insufficiency. His surgical history was remarkable for a bariatric surgery approximately 10 years ago and a right upper lobectomy for squamous cell carcinoma of the lung approximately 2 years ago.

During a follow-up computed tomography (CT) scan for squamous cell carcinoma, a hypodense nodular lesion measuring 48×44 mm was identified at the lower pole of the spleen, suggestive of metastasis. Cemiplimab was initiated as a neoadjuvant treatment while the patient was unfit for surgery. By the time the patient was deemed fit for surgery, the lesion had reduced to 38×31 mm. The case was discussed in the multidisciplinary tumor board and an LSS was scheduled.

## Surgical Technique

### Position and Arrangement of Trocars

Under general anesthesia, the patient is placed in the full right lateral decubitus position, with a block under the costal arch. Pneumoperitoneum is established with a Veress needle. First, a 10 mm trocar is introduced using the open approach on the breast line, 3–4 cm below the costal edge, for a 30° optic. Next, a 5 mm and a 12 mm trocar are introduced under visual control to facilitate triangulation. The 12 mm trocar must be positioned carefully to avoid being too close to the iliac crest. A fourth, additional 5 mm trocar placed in the posterior axillar line is often useful.

### Spleen Dissection

The first step is mobilizing the suspensory ligaments of the spleen and some adhesions that can be found between the spleen and the lower pole. Mobilization of the left colic flexure is rarely necessary. From this lower polar incision, the peritoneum is opened from bottom to top. The dissection continues in the gastrosplenic ligament, to the upper pole, cutting the short vessels as they progress upwards. A fenestrated forceps placed in the fourth trocar can retract the anterior edge of the spleen upward to create more tension.

Surgery continues with careful dissection of the feeding branches of the splenic artery to the inferior pole, where a vessel loop is placed and afterwards divided with a stapler. Once the inferior pole of the spleen is completely mobilized, an ultrasound probe is used to visualize the splenic lesion and mark its boundaries. The devascularized area rapidly changes color and is separated from the healthy parenchyma by a clear demarcation. Indocyanine green fluorescence is used to distinctly differentiate the vascularized zone from the ischemic zone. The parenchyma section is performed in the devascularized area, 1 cm from the borderline, using the COOLINGBIS^©^ radiofrequency device. COOLINGBIS^©^ is a monopolar electrosurgical electrode designed for hemostatic sealing, coagulation, and soft tissue cutting. It uses radiofrequency energy and an internally cooled electrode to facilitate surgical sealing and significantly reduce intraoperative bleeding. Some of this bleeding may occur during this period as the devascularized spleen empties, but it is minimal and ceases quickly. The spleen transection surface may be left as is or covered with hemostatic tissue. The specimen is released into an endobag. Trocars are removed under visual control and the abdominal wall is closed in layers.

## Results

The patient was hospitalized for 3 days and no complications were observed. The hemoglobin post-surgery was 11.2 g/dL, with no further anemization. No signs of hemorrhage were seen. The patient was discharged from hospital and after 2 weeks, a follow-up visit was carried out in the outpatient clinic, with no evidence of postoperative complications. A control CT scan after 1 month only showed a slight amount of intra-abdominal free fluid. The anatomopathological study showed the presence of a nodular area with massive tumor necrosis. There was no evidence of atypical viable cells and the tumor margins were free of disease.

## Discussion

### Benefits

LSS is a rarely used technique but can provide significant advantages beyond those typical of laparoscopic procedures. These advantages include the preservation of immune function, which requires retaining more than 25% of the spleen to prevent post-splenectomy sepsis,^3,10^ as well as the prevention of post-splenectomy splenic vein thrombosis (PSVT). As noted by Lee et al.,^11^ PSVT can occur in up to 45% of cases following total splenectomy, potentially leading to serious long-term complications such as the development of prehepatic portal hypertension. This condition increases the risk of variceal bleeding and other related sequelae, which can significantly affect patient outcomes. Recent insights by Huettl and Lang^12^ underscore the importance of minimally invasive techniques in managing splenic metastases, with emphasis on the balance between oncologic safety and the preservation of splenic function.

### Risks

Nevertheless, subtotal splenectomy has some pitfalls, including technical difficulties, particularly with transection of the splenic parenchyma. It is not applicable to central cysts with hilar involvement or very large cysts. Additionally, there is a risk of recurrence in hematological diseases and a theoretical increase in morbidity due to intraoperative complications, especially hemorrhagic complications, although not all series report such outcomes.^3,7^ Therefore, there is a need for advanced hemostatic technology, such as a radiofrequency device. COOLINGBIS^©^ is a valuable instrument for controlling hemostasis and managing bleeding from the spleen during surgery. Presurgical preparation is crucial, involving the identification of the vascular pedicle on a CT scan and the use of indocyanine green to visualize spleen vascularization and delimit ischemic zones.

### Potential Indications

Another point is the fact that metastatic lesions are not typically a clear indication for partial splenectomy.^8,10,11^ Given the right circumstances, we believe that metastatic lesions, although a relatively uncommon occurrence, could be an indication for subtotal splenectomy. In this case, we consider it an appropriate surgical approach given that the patient only had one metastatic lesion, the lesion measured < 50% of the spleen and was in an appropriate location (lower pole of the spleen), and the lesion had a partial response to neoadjuvant treatment. Taking into account all these factors, we choose a partial splenectomy over a total splenectomy, with all the advantages it entails.

In the study by Vega et al.,^13^ the authors’ results aligned closely with those observed in our research, particularly in the effective use of LSS for managing tumorous lesions. Both studies highlight the safety and feasibility of this approach, even in cases of unknown primary cancer; however, they also underscore an important limitation that our study also shares—the short follow-up period after surgery. While our immediate postoperative outcomes are promising, including low morbidity and effective tumor resection, the lack of long-term follow-up data precludes us from making predictions about long-term survival or recurrence rates. Consequently, further studies with extended follow-up are necessary to validate these conclusions and better understand the long-term implications of LSS.

## Conclusions

In selective cases, LSS is feasible and safe when performed with the appropriate laparoscopic equipment and by surgeons with extensive experience in laparoscopy.

## Supplementary Information

Below is the link to the electronic supplementary material.

Supplementary file1 (MP4 154388 KB)
